# Here it is everything at once: a qualitative study of district nurses’ experiences caring for older individuals with multiple chronic health problems in primary care

**DOI:** 10.1186/s12875-026-03574-1

**Published:** 2026-10-09

**Authors:** Annica Lagerin, Elisabeth Bos Sparén, Caroline Wachtler

**Affiliations:** 1 Department of Health Care Sciences, Marie Cederschiold University, Stockholm, Sweden; 2 Department of Neurobiology, Care Sciences and Society, Division of Family Medicine and Primary Care, Karolinska Institutet, Huddinge, Sweden

**Keywords:** District nurses, Mental health, Multimorbidity, Older people, Person-centered care, Qualitative research.

## Abstract

**Background:**

Over the next 10-year period in Sweden, the number of people aged 80 and older is will increase by about 50%. The care needs of the population are changing as more people live at home longer with chronic conditions. District nurses in primary care often have the first contact with patients of all ages and are especially involved in care of older individuals with multiple chronic conditions, common mental health problems, and complex care needs. Understanding how district nurses currently see their work with older people with complex multimorbidity is a key step in developing new models of primary care to support older individuals with complex multimorbidity. The aim of this study was to investigate district nurses’ experiences of caring for older adults with multiple chronic conditions and how they think care be improved for this group.

**Methods:**

This was a qualitative study of individual and focus group interviews made with fifteen district nurses working at five primary care centers in the Stockholm Region, Sweden. Data was analyzed using reflective thematic analysis.

**Results:**

We generated one central theme, Ready to take responsibility if there is room for complexity and four subthemes: Seeing the complex patient as a coherent and whole person; Competent to take responsibility for patient needs.; Primary care can’t say no, so DNs find solutions when cooperation is lacking; DN competencies must be visible, well-defined and have boundaries.

**Conclusions:**

DNs feel competent to identify and address individual needs for older people with multiple chronic conditions and common mental health problems and ready to take responsibility for patient’s needs, if they have the time, support and structure needed to address complexity with this patient group.

## Background

The care needs of the population are changing as more people live at home longer with chronic conditions. It is now normal that people live a long time with many health conditions [1]. Over the next 10-year period in Sweden, the number of people aged 80 and older is projected to increase by about 50% [2]. Multimorbidity is often defined as having two or more chronic conditions, but as chronic conditions accumulate both biological patient complexity increases due to accumulation of disease burden, and psychosocial patient complexity increases as treatment burden and illness symptoms affect the individual’s life and social network [3]. Risk for multimorbidity increases with age [4–6]. Additionally, risk for mental disorders like depressive symptoms, worry, sleep problems and anxiety increase for people over the age of 65 with multiple chronic diseases [6–8]. People with complex multimorbidity, meaning multiple physical and mental disorders, often have increased care needs [3]. The current demographic shift requires new solutions to provide person-centered care for older individuals with complex multimorbidity to address the diagnoses a person has, and to provide care that considers the individual’s particular situation, expectations and needs.

Primary care serves as a central hub for delivering person-centered care to older adults with complex multimorbidity, coordinating across health, medical, and social services. Person-centered care emphasizes responding to each individual’s narrative and priorities and documenting their insights, wishes, and responsibilities to guide care planning—a principle shown to improve quality of life and reduce healthcare utilization among older adults with multimorbidity [9]. In Sweden, primary care aims to provide accessible, coordinated care that supports health, optimizes resource use, and enables older adults to age safely at home, reflecting their strong preference to remain in familiar environments [10–12]. Despite primary care's cornerstone role in health systems, significant gaps persist in achieving truly person-centered practice. Primary care professionals often describe their work as a complex balancing act—navigating patient needs, professional ideals, practical constraints, and emotional challenges—while striving to avoid low-value care and minimize disruptions to patients’ lives [13]). Addressing these gaps is essential to improve outcomes, enhance patient experience, and reduce costs [14].

District nurses (DNs) in Swedish primary care are uniquely placed to support development of new primary care models for person-centered care of older individuals with complex multimorbidity. The DN role is generalist, with focus on holistic care (for more information on the role, see Box 1 [15–17]). The interaction between DN and an older person in primary health care can be understood as a complex social process in which the nurse navigates the balance between personal and professional roles, integrates person-centered and task-oriented approaches, and applies both salutogenic and pathogenic perspectives [18]. Community nurse-led interventions to support care transitions, self-management and integrated collaborative care for the general population have shown effectiveness for improving a range of patient outcomes including physical function, chronic disease management capacity, medication adherence and knowledge, and perceptions of service quality and coordination [19]. An previous Swedish study indicates that DNs use of tools like Safe Medication Assessment (SMA) and clinical decision support systems improves medication safety for older adults, indirectly supporting mental health by reducing stress and confusion related to polypharmacy [20].

### District nurses (DNs) in Swedish primary care are specialist nurses with advanced training in community and home-based care

- 75 European Credit Transfer System credits and prescribing rights for certain medications, incontinence and diabetes aids.
- Trained to provide holistic care to people of all ages in the community.
- Specialists in health promotion such as tobacco cessation, dietary advice, sleep, alcohol abuse, and issues related to stress and mental illness.
- First point of contact for older individuals with multiple chronic conditions, common mental health problems, and complex care needs.
- Providers of continuous, coordinated care to patients with chronic diseases.
- Educators about self-care for patients and their families.
- Providers of nursing care in the home.
- Team members at the primary care center with general practitioners (GPs), registered nurses, and assistant nurses.
- Responsible for coordinated care plans for patients with chronic diseases who need interventions from health, medical care, social services.

### Box 1. Roles of the district nurse in Swedish primary care

Understanding how DNs currently experience their work with older people with complex multimorbidity and what they think can be improved is a key step in developing new models of primary care to support older individuals with complex multimorbidity. The aim of this study was to investigate DNs experiences of caring for older adults with complex multimorbidity and how they think care be improved for this group.

## Methods

### Study design

In this qualitative study we aimed to examine DNs experiences of caring for older adults with complex multimorbity including multiple physical and mental disorders, using reflexive thematic analysis (RTA) of individual interviews and focus groups with DNs working in primary care centers in Region Stockholm, Sweden. We chose RTA as an appropriate approach because our research question is explorative, broad, and particularly focused on understanding how participants themselves make meaning of their experiences [21, 22]. Themes generated in RTA can be used to inform further development of care models or interventions [23, 24].The analysis and reporting followed the Reflexive Thematic Analysis Reporting Guidelines (RTARG) [25].

### Approach to the research

Our research team brought clinical experience to the project. AL and EBS are both DNs with many years of clinical experience with older patients in primary care. CW is a primary care physician with active clinical practice. We are all experienced qualitative researchers. Our clinical experience forms our pre-understanding of what constitutes good patient care and gives us a biopsychosocial perspective on illness and disease. However, there is also some tension between our clinical roles as physicians and nurses traditionally have a power differential in the clinic, where physician medical knowledge and decision-making usually is given higher status than DN patient care. We expected our clinical experience to influence both data collection and analysis, as we have a deep understanding of working in the same context as participants. In addition, we are all part of a research network committed to improving care for older people with multiple chronic conditions in PCC. We bring an intervention and clinical problem-solving lens to the analysis. Two authors (EBS and AL) identify culturally as Swedish, while CW is raised in the USA.

Epistemologically, we took a constructivist perspective to the analysis, with an understanding of knowledge constructed through individual lived experiences and awareness that our lived experiences as researchers and clinicians influence the knowledge we generate in interaction with our research participants and data [26]. Ontologically, we position ourselves as interpretivist, understanding truth as contextualized, in contrast to a positivist position from which truth is the result of unbiased, objective measurements [27].

### Setting, sampling and recruitment

The study was conducted in Region Stockholm, Sweden. The region has a population of 2 480 000 served by 250 PCCs varying in size between 1000 and 35,000 listed patients [28]. There are 14,000 licensed DNs in Sweden, and 11,500 are actively employed [29]. Approximately 2880 DNs currently work actively as DNs at PHCCs, in community care for elderly people and as school nurses in Region Stockholm.

Participants were recruited using purposive sampling. First, we contacted a professional network for DNs and presented information about the study aim and background of the research team at a meeting of the network at which interested individuals were asked to directly contact the researchers. AL and EBS are members of this network and know some of the participants professionally. Via DNs who expressed interest, we contacted PCCs at which they worked with the aim of recruiting participants from PCCs with catchment areas with differing socioeconomic backgrounds, because primary care can be structured differently depending on the size of practice and the nature of the population that is served as well as the municipality the center is located in. We used the neighborhood social deprivation as measured by the Care Need Index to guide this purposive sampling [30]. We contacted 8 practices in total, and 5 of these helped us recruit participants, while three were unable to help despite interest due to low availability of personnel and time constraints. We had the original intention of conducting focus groups, and recruited 17 DNs in total, of which 15 participated. One focus group was converted to a one-to-one interview due to illness, and but the other 4 focus groups each included 2–5 participants.

### Dataset generation

The one-on-one interview and the focus group interviews were conducted by AL and EBS between May 2023 and September 2023. After a few questions about participants’ backgrounds and years of experience in primary care, a semi-structured interview guide was used to ensure the interviews covered the following areas of interest:

- what is it like to work with individuals with physical and mental disorders?
- how do you identify patients who need more care and support?
- how do you experience communication and collaboration with other healthcare professionals and other professions?
- what would you like to change about how you work with this patient group?
- how could care be improved for these patients?

Interviews did not follow a standardized script but were rather conducted as an open discussion guided by the topic guide. Interviews were held at the DNs´ workplace. They took between 40 and 60 min and were digitally recorded and transcribed verbatim in Swedish by a company (trankriberingshuset.se). Transcripts were pseudonymized for confidentiality. Field notes regarding participants information and reflections on the interviews collected as well in accordance with the method [31].

During data collection, the research group held regular meetings to discuss recruitment and data collection. Summary notes were written after each meeting. The group discussed information power from the interview material and came to a consensus to stop data collection when the information power from the data was considered adequate by all group members [32].

### Data analysis

Data analysis was done using six-phase reflexive thematic analysis [22]. All data from individual interviews and focus groups as well as field notes were included in the dataset and we used RTA to conduct cross-case analysis, comparing and contrasting data within and between interviews and fieldnotes. RTA is an inductive approach in which themes are generated based on the comparing and contrasting the collected data rather than fitting data into a pre-existing theoretical framework [21]. In the analysis process, we handled data digitally using the NVivo Program (version 20.7.2.1560) for Windows qualitative analysis software, Roam Research (https://roamresearch.com/), by writing and revising, and by creating thematic maps of proposed themes and subthemes using Microsoft Word and PowerPoint. Participants were not included in the analysis process.

In the first phase of analysis, all authors read the entire data set. Authors then met to discuss initial reflections on the data set; CW wrote memos during this process. In the second phase, coding, AL and EBS worked together to code interesting features in the text for the entire dataset in NVIVO, while CW coded the data independently using Roam Research (https://roamresearch.com/). In the third phase, the team met to generate the initial themes based on relationships between codes. At this point, the team lifted and discussed differences and similarities in how individuals had coded the text, and used this discussion to revise some codes, rename some codes, and to inform them. Discrepancies in how researchers coded text were especially explored, as these differences were seen to reflect differences in researcher pre-understanding and positioning and we wanted to lift these different perspectives in the analysis. CW documented this process in memos by collating codes and gave potential subthemes descriptive names. In the fourth phase, CW led the team in reviewing and defining potential themes and subthemes. In the fifth phase, the overarching theme and four subthemes were named and defined in an iterative reciprocal reading process involving all authors, who re-read the interview text and memos to check that the theme and subthemes worked in relation to the coded text and the entire data set. At this point, the language used for analysis was changed to English. In the final phase of analysis, all authors contributed to write-up in an iterative process. All included citations were translated into English by CW, a native English speaker.

### Ethical considerations

Participants were given written and oral information about the study´s aims and the study was approved by the Swedish Ethical Review Authority (2019–05018).

## Results

A total of 15 DNs, 14 women and one man, participated in this study. Participants were between 31 and 64 years of age. Their professional experience as DNs ranged between 1 and 25 years (median 13 years). Participants worked in different geographic areas both rural and urban in the Stockholm Region, varying in CNI between 0.53 and 1.44 at the time of data collection.

The central theme generated in the analysis was *Ready to take responsibility if there is room for complexity*. The DNs described complexity in their work as depending on several different factors that work together to make health care demanding for some patients: patient factors like multiple chronic physical and mental disorders and polypharmacy, the structure and limits of the primary care clinic and health care organization, and needing to manage communication and cooperation within and outside of health care.

DNs in this study expressed that they saw responsibility for older patients with multiple chronic conditions as central to their professional role. They have professional engagement, a way of working that considers the whole person, and the practical skills to take responsibility for these patients.

*“This is truly an amazing group with so much competence*,* but it’s almost at the limit—I think we would need a bit more resources*,* really”. (Group 2*,* DN 9)*

They had concrete suggestions for how they could take on this responsibility, reflecting a “readiness” to do the work if there is room for it in the health care structure.

*“To catch their mental health problems and their needs*,* continuity is extremely important*,* building a relationship with them*,* somebody who has responsibility for the social aspects. What is the family structure like? What kind of help do they have from family and friends*,* to be able to see the problem from different perspectives. And then maybe start addressing one problem at a time. Some*,* I have some who are just like*,* here it is everything at once maybe five at a time*,* and they can’t do it. It is maybe important to take one thing at a time and be calm”. (Group 1*,* DN 2)*

We generated four interrelated subthemes that together make up the central theme. DNs described *seeing the complex patient as a coherent and whole person *and this made them 2. *competent to take responsibility for patient needs.* However, this responsibility could grow unchecked, as *primary care can’t say no, so DNs find solutions when cooperation is lacking*. To maintain balance and capacity *DN competencies must be visible and defined with boundaries*.

### Seeing the complex patient as a coherent and whole person

DNs understood their patients as individuals in which the complexity of multiple medical conditions, medications, age, social situation, and care needs all contribute to the individual’s problems. Seeing the patient as a coherent, whole person, rather than accumulation of chronic medical conditions, was seen as central to the DN role. DNs did report that patients most in need were often older and very ill, and that this made the medical challenges of their work more difficult, something that could be alleviated by continuity.

*“I think they are more demanding*,* or more in need of resources in the sense that we have to have a lot of knowledge about medicine*,* many diseases because they are multimorbid*,* and we always have to have our clinical judgement with us we meet them to be able to know if it is one of their diseases that is getting worse or what is normal for that disease and so on. So*,* we have to get to know them better and better and that is easier when you have continuity actually”. (Group 1*,* DN 2)*

Seeing the patient as coherent and whole depended on shifting focus away from the DN agenda and towards the patient, something that could be challenging due to medical complexity.

*“I think sometimes*,* we forget to ask the patient what they want*,* how they feel. There is so much you can do all the time to help*,* but on the other hand*,* what does the patient want? Sometimes you see a patient who comes*,* and you think*,* wow that is a big problem but the patient just wants to talk about their mental health or something*,* and that is their biggest problem. . You must find the right focus for the patient so that they are participating and want to*,* and that is a challenge. Often you want to do so much yourself*,* but what does the patient*,* the person*,* actually want?”. (Group 1*,* DN 1)*

Seeing the patient as coherent and whole could involve communication with family members and considering cultural context.

*“Sometimes they have a relative with them. We have a lot of people from other cultures*,* so we have a lot of interpreted visits*,* and they often have their children with them”. (Group 5*,* DN 15)*

DNs expressed pride when they could work with the whole person and provide continuity of care, and thought this way of working led to better care.

*“I think being to be able to identify mental disorders and their needs*,* it is so important with continuity*,* building a relationship with them*,* so you maybe take more responsibility to consider the social issues around them”. (Group 1*,* DN 1)*

DNs also felt working in a way that preserved the patient as a coherent and whole person was a way to ensure equitable care. Often, the most complex patients had the least resources to actively ask for what they needed, so DN work to explore patient needs was a way to address this.

*“Those most in need of care never ask for it”. (Group 4*,* DN 14)*

One suggestion the DNs discussed to provide care for the complex patient as a coherent and whole person was development of Elderly Care Units at all PHCs in Region Stockholm, open at least one hour a day for appointments, an open telephone line for elderly, and development of preventive home visits for older people by DNs.

*“I think that an Elderly Care Unit*,* one that functions*,* and could be open all the time*,* could even reduce the burden on physicians”. (Group 5*,* DN 15)*

### Competent to take responsibility for patient needs

DNs described their competency for both identification of patients with multiple complex needs, and for addressing these needs. They have many opportunities to identify and assess patients: during visits, when they had contact with patients’ relatives, children, or neighbors, or via the Elderly open telephone line. Furthermore, GPs and healthcare professionals at hospitals contacted DNs when an older patient needed help. They described patients with complex needs with many different illnesses and health-related problems, including cardiovascular disease, COPD, diabetes, and dementia. DNs felt in particular that mental disorders gave rise to complexity, and patients with complexity were more in need of DN competencies. DNs described often identifying loneliness among the elderly as a cause anxiety and sadness.

*“I think many people who are homebound get depressed because of their chronic illness. I think there are even some younger people who don’t have a personal assistant who are chained in place at home*,* they feel awful”. (Group 3*,* DN 10)*

DNs identified some older patients who risked receiving lower quality care than younger patients, for example in contact with specialist clinics, or when the elderly in HHC found it difficult to leave their homes due to mobility difficulties.

*“I have some people where*,* yes they are older*,* but they are so energetic and fresh and have ambition and want to have their insulin and so on – but they don’t get the same help because it’s ok to be 80 and then we turn a blind eye. I don’t think that’s ok”. (Group 1*,* DN 3)*

DNs identified and assessed patients, and then also took responsibility for helping the individual until the problems were solved.

*“Often*,* we are their voice. We meet them at home or at the clinic*,* they tell us something*,* and we carry it until we have solved the problem. That’s usually how it works*,* we try to find solutions”. (Group 1*,* DN 4)*

DNs expressed confidence in their competency for caring for this patient group. They felt they have good knowledge of diseases and medications.

*“You need a lot of knowledge around medications and diseases because they have multimorbidity*,* and we always have that clinical judgement with us when we meet them in order to know if this is one of their diseases that is starting to get worse or what is normal for the disease and so on”. (Group 1*,* DN 3)*

DNs felt support from their team members at the primary care center and often took on the role of supervisor for colleagues.

*” And you come in earlier so you can take care of your administration and still have the door open. It’s mainly the home healthcare team that brings up various tricky issues and what they’re thinking about or worried about to you. I think that’s great ”. (Group 4*,* DN 13)*

To improve their potential to take responsibility for assessing and addressing patient needs, DNs suggested improving continuity of care. For example, they proposed a limit to the number of home health care (HHC) patients so one DN could be responsible for a maximun of 35 individuals. Additionally, they proposed that some older complex patients should have a contact person who could coordinate their healthcare needs. Finally, DNs identified a need for supervision and mentorship to support them in assessing and addressing patient needs, especially with more complex patients.

*“Debriefing in my office every morning*,* I don’t know if it counts as supervision”. (Group 4*,* DN 12)*

### Primary care can’t say no, so DNs find solutions when cooperation is lacking

DNs felt that because the needs of the complex patients were so immediate, they often made medical assessments and solved difficult problems on their own, even when this was stressful or draining, without adequate support. DNs felt primary care couldn’t say no to the patients, but that it was difficult to provide medical care because cooperation between different health care providers and well as between providers and the municipality was lacking. DNs had a sense of responsibility for patients, and therefore tried to find individual solutions each time this kind of problem came up. One example of this situation was when individuals were sent home from hospital without any prior communication from hospital staff.

*“It’s just*,* bang now they are going home and then everything needs to be in place”. (Group 5*,* DN 15)*

In this kind of situation, DNs would do their best to make sure the individual could be received at home despite not having time to prepare or plan.

Additionally, collaboration with municipality staff like assistance administrators and home care staff often felt inadequate. It could take a long time before DNs received information about which home care group the patient had hired. Many DNs said they collaborated with 7–10 different home care groups, which complicated care for older patients. Sometimes, lack of collaboration was seen as related to lack of knowledge.

*“I had chronic care planning last week with a couple who were 98 and 98 years old and the assistance administrator thought that the amount of time they had home care a day should just be increased first without thinking about how multimorbid they are. That might not be enough just for the home help to come one more time for them to get the right support. I think they don’t understand how sick they are*,* people who live at home and have care from primary care”. (Group 2*,* DN 8)*

DNs identified that patients with complex multimorbidity often turned to DNs for support and for help solving problems, even if the problems needed solutions outside of health care.

*“We can’t do everything for them*,* but they maybe come to us because they don’t know where to turn in the first place*,* and maybe it is more socioeconomic problems that an assistance administrator has to be more involved in for them to be helped than something we can do*,* like for example if it’s about supported living”. (Group 1*,* DN 3)*

DNs experienced there was no room in the current economic model for cooperation between different actors at the primary care level even though it would have benefited patients.

*“It is hard to make it work; it was easier before. We had more cooperation then; rehab could also come. We had meeting once a month and discussed our shared patients that needed a lot from all of us. It was really good*,* but also one of those contracts*,* no*,* we don’t have time for that. It’s like time costs money*,* I mean*,* you have to be paid for the time you use for the services you do*,* and meetings don’t count”. (Group 3*,* DN 11)*

DNs described that they felt strong collegial support at their PCCs and were able to access this network to solve problems for the patients.

*“We have each other*,* and we really take advantage of each other’s knowledge and ask about resources”. (Group 1*,* DN 4).*

Many PCCs had specialized care clinics for patients with asthma/COPD, diabetes and incontinence led by registered nurses and this cooperation facilitated DN’s care work.

DNs suggested that creating more opportunities for patients to have home health care, and setting aside time expressly for cooperation could improve cooperation between DNs, the municipality and hospitals. They suggested that more time for cooperation with the municipality could help improve home care staff knowledge about chronic diseases. DNs also had suggestions for improved cooperation in the primary care clinic, for example by using planning days at the center to improve communication about caring for patients with multimorbidity, to work on ways to improve relational continuity and to improve early identification of mental illness in this group.

### DN competencies must be recognized and defined with boundaries

The DNs perceived they had good competence in nursing care for patients with multimorbidity, but the health care organization often did not recognize or fully access their broad knowledge and competencies. In addition, the DNs themselves often did things for patients that were outside the limits of nursing care to compensate for what they saw as system failures. DNs expressed that when they could use their competency in continuity, care for older individuals improved. However, DNs did not feel that their role was recognized by leadership.

*“Now we have a chief who isn’t medically trained in that way. Our highest chief*,* so you can’t really turn to them either. Then we have another chief who isn’t ether a DN or a nurse. I don’t think that person really sees the whole picture and instead only sees the telephone*,* the responsibility we have there and doesn’t see how we others work”. (Group 5*,* DN 15)*

DNs suggested that it was the responsibility of leadership to be informed about the DN role, and to recognize and support the potential of the DN role to improve care for older individuals. For examples, DNs could be involved in planning of work processes, and other staff could be informed of DN competencies.

The DNs described trying to provide quality care despite systemic problems, something that often took a toll in energy and time, leading to stress and feeling they could not do their work with quality. They described for example double checking that other professions were doing their job, for example following up test results. At some centers, temporary staff were hired, with lower competency level than ordinary staff. In some centers, premises were cramped. Nursing care required time, work materials and collaboration with other health care professions as well as time for follow-up care and treatment. Despite feeling lack of time, DNs prioritized care provision.

*“We have a framework*,* what the work is like*,* work hours and so on*,* but we make priorities in everything we do*,* and sometimes we don’t have the time for it*,* but we do it anyway”. (Group 2*,* DN 7)*

They expressed that it was especially difficult to decide how to set limits for care provision for the individual patient.

*“Where is the limit and when should you give in and have the possibility to provide less help. These types of questions are very difficult. . because we are so ambitious. We are nurses by nature*,* and you see a need and you want to fix it*,* you know someone else could also do it but you pull out all the stops*,* write referrals*,* try to make it better*,* we think. Yeah*,* it is really hard*,* setting a limit”. (Group 3*,* DN 10)*

DNs recognized that especially patients with mental disorders sometimes needed professional support beyond what DNs could offer.

*“Yeah*,* we act like psychologists*,* in quotation marks. I mean*,* you try to do your best and be there for support and conversation*,* but we don’t have that training and especially we don’t have the time”. (Group 3*,* DN 11)*

Setting boundaries around how much responsibility to take for patients was recognized as a challenge.

*“We can have a hard time ourselves shifting work over to others”. (Group 3*,* DN 11)*

The DNs drew on each other for collegial support in relation to patient care, but they highlighted that collaboration with physicians and other medical professionals was vital for maintaining dialogue about patients’ treatment. In some units’ practices, DNs reported that communication with physicians was already working very well. However, they thought being able to work with physicians in a team would be a way to lessen stress and worry in their work.

*“It would be nice to have closer access*,* especially to the doctor who could also be able to come out and see the patient*,* and that we could discuss things there.” (Group 3*,* DN 10)*.

For patients with mental disorders, DNs suggested better access to health care professionals with competency for mental health.

*“The ideal situation would be to have a social worker or someone with the right training who we could contact and who could actually make home visits to these patients. Very often you feel*,* well*,* inadequate*,* and I mean*,* they really can’t get anywhere themselves*,* the home care patients. They are the ones affected by all sorts of things*,* as you said—grief*,* anxiety*,* or*,* yes*,* mental health issues.” (Group 3*,* DN 11)*.

## Discussion

District nurses described their work with older adults with complex multimorbidity as demanding but central to their professional role, emphasizing a strong sense of responsibility and readiness to provide person-centered care when organizational structures allow. They highlighted the importance of continuity, relationship-building, and addressing both medical and social needs, while noting that mental health issues often add complexity. DNs felt competent to identify and manage these needs but reported systemic barriers, such as fragmented collaboration with hospitals and municipalities, lack of time, and insufficient recognition of their expertise. To improve care, they suggested structural changes including Elderly Care Units, limits on caseloads, designated contact persons for complex patients, better teamwork with physicians, and access to mental health professionals for home visits. They stressed that defining DN competencies and creating space for interprofessional cooperation are essential for sustainable, high-quality care for this patient group.

The central theme generated in our analysis, *Ready to take responsibility if there is room for complexity*, reflects DNs’ strong professional commitment to managing complexity when organizational structures allow. This readiness aligns with previous research emphasizing that structural support is essential for nurses to deliver person-centered care in home settings [33, 34]. Our findings also echo earlier studies showing that DNs often compensate for systemic gaps, which can lead to ethical stress and role ambiguity [35, 36]. The tension between professional ambition and organizational limitations underscores the need for reforms that clarify roles and strengthen interprofessional collaboration to maintain care quality and patient safety [37, 38].

DNs in this study emphasized *Seeing the complex patient as a coherent and whole person*, an approach that integrates medical, social, and psychological dimensions of care. They described continuity and relationship-building as critical for managing complexity, particularly when mental health issues were present. This finding resonates with previous research showing that person-centered care improves patient satisfaction and outcomes [39, 40]. Mental disorders were identified as central to patient well-being, similarly to literature that links such conditions in multimorbid older adults to increased mortality and reduced quality of life [41]. However, time pressures and organizational barriers often hindered this approach, echoing earlier findings that nurses risk missing patient priorities when schedules are too tight [34]. Strengthening continuity and communication within primary care could therefore enhance equity and responsiveness for this vulnerable group.

Participants described feeling *Competent to take responsibility for patient needs*. This is consistent with literature recognizing DNs’ competency in managing multimorbidity and mental health concerns [36, 42, 43]. Our findings also reveal that DNs often act as coordinators, bridging gaps between patients and fragmented health systems—a role previously identified as vital for continuity of care [44]. However, lack of supervision and mentorship was noted as a barrier, reflecting earlier studies that link insufficient support to stress and reduced care quality [45]. Suggestions such as limiting caseloads and assigning care coordinators align with evidence that structural adjustments can mitigate burnout and improve patient safety [38, 46, 47]. These findings highlight the importance of organizational strategies that enable DNs to fully utilize their competencies while maintaining professional balance.

DNs experienced that *Primary care can’t say no*,* so DNs find solutions when cooperation is lacking.* This finding is mirrored in prior research on care transitions and coordination failures [37, 48]. This lack of systemic integration forces nurses to create ad hoc solutions, increasing workload and stress—issues well documented in studies of home health care [37, 49, 50]. Our participants’ suggestions for planning days, improved communication, and dedicated time for interprofessional meetings reflect recommendations in earlier literature for structured collaboration to enhance continuity and reduce ethical strain [51]. Addressing these gaps is critical, as poor coordination has been shown to compromise patient outcomes and nurse wellbeing [52, 53]. Organizational models that prioritize time for collaboration and shared responsibility could significantly improve care for older adults with multimorbidity.

While DNs expressed confidence in their clinical skills, they felt *DN competencies must be recognized and defined with boundaries*. This aligns with previous findings that misconceptions about DN responsibilities create barriers to effective discharge planning and patient relationships [37]. Our study also highlights ethical stress arising from unclear boundaries, echoing literature on the challenges of reconciling competing expectations in community care [35, 47, 54]. Participants’ calls for organizational recognition, involvement in planning, and clearer role definitions are supported by evidence that such measures reduce burnout and improve job satisfaction [46, 47, 55]. Without these changes, nurses risk overextending themselves, compromising both their wellbeing and the quality of care delivered. Defining boundaries and promoting role clarity are therefore essential steps toward sustainable care models.

### Strengths and limitations

This study has both strengths and limitations. We applied reflexive thematic analysis, a method well suited for exploring complex research questions and deepening understanding of lived experiences [21]. Using focus groups enabled interaction among participants, which often generates richer insights into shared phenomena, opinions, and experiences [31]. All members of the research team have clinical experience in primary care for older patients, which may have facilitated participant recruitment as two members of the team were DNs themselves and could be seen positively and as colleages by potential participants. Our insider knowledge may have informed follow-up questions in the focus groups and the analysis that enriched the study. In our purposive sampling, we recruited DNs who worked at centers with varying socioeconomic backgrounds and sizes to reflect varying working conditions depending on the size of practice and the nature of the population that is served as well as the municipality the center is located in. This approach may have given a wider range of experiences than if we had only recruited from one practice, or from practices that were similar to each other. To guide sample size, we used the concept of information power, appropriate for reflexive thematic analysis [56]. Our narrow research focus, purposive sampling of participants with extensive knowledge of primary care for older adults—including those working in varied socioeconomic contexts—combined with the focus group approach and cross-case analysis, ensured that the data contained sufficient information power to address the research question.

However, the insider perspective of the research team may also be a limitation. While clinical experience enriched interpretation, including a researcher with a different professional background might have provided additional depth and alternative perspectives. Furthermore, the study could have benefited from purposive sampling to include participants with varying lengths of experience or from primary health care centers with different organizational strategies. These approaches were beyond the scope of the present study but represent opportunities for future research.

### Implications

This study provides concrete examples of how district nurses propose addressing care needs for an aging population with multiple chronic conditions and common mental health problems. They recommend establishing clear standards for the organization and functions of Elderly Care Units. These units should be accessible to the population and allow DNs sufficient time to meet individual needs. A person-centered approach with relational continuity should be central to these units, alongside strong collaboration with municipalities and other healthcare actors. Our findings also highlight that the current work environment for DNs is not sustainable, as DNs currently often work to compensate for systemic gaps without adequate recognition or boundaries. The balance between responsibility and organizational capacity for complexity needs to be addressed if DNs are to provide quality care over the long term. The DN role must be clearly defined within primary care and their contributions made visible. Additionally, DNs need dedicated time and support from leadership for structured reflection and collegial dialogue, to improve patient care and develop strategies that prevent fatigue and burnout.

This study also points to important areas for future research. There is a need to explore patients’ perspectives on receiving home care or care from DNs. Furthermore, evaluating the impact of organizational changes on care quality for the aging population will be essential to inform sustainable models of primary care.

### Conclusion

District nurses are essential for providing holistic care to older adults with complex multimorbidity, but systemic barriers such as fragmented collaboration, unclear role recognition, and time constraints limit their capacity. Organizational changes that clarify DN roles, strengthen teamwork, and integrate mental health and social support into primary care are critical to improving care quality and supporting nurse wellbeing.

## Data Availability

The datasets used and analysed during the current study are available from the corresponding author on reasonable request.
